# Barriers and facilitators to mental health care experienced by youth involved in child welfare and their caregivers

**DOI:** 10.3389/fped.2026.1763516

**Published:** 2026-04-20

**Authors:** Christelle Tan, Courtney Dunn, Katie Fox, Houley Sall, Sarah Beal, Mary Greiner

**Affiliations:** 1 Department of Psychiatry, Cincinnati Children’s Hospital Medical Center, Cincinnati, OH, United States; 2 Department of Psychiatry and Behavioral Neuroscience, University of Cincinnati College of Medicine, Cincinnati, OH, United States; 3 Comprehensive Health Evaluations for Cincinnati's Kids (CHECK) Center, Cincinnati Children's Hospital Medical Center, Cincinnati, OH, United States; 4 Department of Pediatrics, University of Cincinnati College of Medicine, Cincinnati, OH, United States

**Keywords:** caregiver perspectives, child welfare, mental health service accessibility, qualitative analysis, youth perspectives

## Abstract

**Introduction:**

Child welfare involved youth have high rates of behavioral health needs; however, many never receive needed mental health services. The specific barriers children and their caregivers face are not well described. The objective of this study was to describe the process and experience of initiating mental health care for youth entering out-of-home care.

**Methods:**

Youth-caregiver dyads were enrolled in a 12-month longitudinal study, after entering protective custody for the first time, during which dyads completed surveys every 3 months. Electronic health records data linked with administrative child welfare data were extracted. Subgroups of dyads were identified based on reported mental health needs and whether needs were met over time. Descriptive analyses of these subgroups were completed to compare sociodemographic and child welfare characteristics and mental health screening scores. Youth or caregivers of youth with reported unmet mental health needs were recruited to participate in semi-structured interviews regarding experiences seeking mental health services. Timeline recall was used to support enhanced reporting. Timelines created during interviews were analyzed to uncover additional patterns in the events described. Thematic content analysis was applied to all interviews using Dedoose.

**Results:**

*N* = 201 youth-caregiver dyads were enrolled in the longitudinal study. The survey data showed that most youth entering out-of-home care have mental health needs, and these needs were inconsistently met over time. Descriptive analysis of subgroups showed that most characteristics were comparable across groups; however, some differences were noted. Nineteen dyads completed the interview. Timeline analysis revealed that adolescents rarely access mental health services prior to child welfare involvement, and caregivers who recognized service need and acted early accessed resources quicker than those who did not. Thematic analysis revealed both barriers and facilitators to mental health care which manifested in three primary categories: Caregiver, youth, and family-of-origin attributes, the mental health system, and other social service systems.

**Conclusion:**

Most youth access mental health services while in out-of-home placement; however, mental health needs are often not consistently met over time. Caregivers play an important role in both reporting mental health concerns and accessing mental health services. Integrated mental health services are an important resource for child welfare involved youth seeking mental health treatment.

## 1 Introduction

Mental health service utilization among youth with child welfare involvement is complex. Youth in child protective custody (e.g., foster care) have high rates of behavioral health needs and utilize mental health services 5–8 times the rate of Medicaid-insured youth not in child protective custody (1). Furthermore, outpatient mental health service utilization varies over time, with a rapid increase in mental health service use documented following an investigation, especially among youth who are placed in out-of-home care (2). Despite these findings indicating high rates of mental health utilization among children with child welfare involvement, studies show that less than half of youth with behavioral health needs ever receive services (1). While child welfare involved youth in out-of-home placements are more likely to access mental health services than children who remain at home, many youth in out-of-home care still do not access needed mental health services (3).

Barriers to mental health care services have been described and include frequent change of placement, inconsistent mental health assessments, difficulty communicating across systems, limited training for caseworkers, caregivers, and healthcare providers in the identification and treatment of mental health disorders, and insufficient mental health service availability (4–6). However, most of the existing literature describes these barriers from the perspective of professionals involved in the coordination of care for children, including case workers and healthcare professionals. The specific barriers children and their caregivers face as they seek mental health care while in child protective custody and out-of-home care are not well described in the literature.

From a social-ecological framework, youth and caregiver perspectives would strengthen our understanding of how individual level barriers intersect with community and policy level constraints and how those interactions shape access. This knowledge is necessary to develop strategies that are helpful and practical for caregivers and youth seeking mental health services. This study leveraged a mixed methods approach to comprehensively describe existing barriers to and utilization of mental health services among youth in out-of-home placement. First, we conducted a descriptive analysis of survey data collected from a subsample of participants represented in the larger administrative dataset to better understand how youth mental health needs were met overtime, integrating participant reports with administrative records. Second, we completed a timeline analysis of interviews with youth and their caregivers (all of whom are also represented in the survey and administrative data) to identify patterns of mental health service use shared by participants that capture events beyond those reflected in the administrative record. Finally, we did a qualitative analysis of interviews to describe the process and experience of accessing mental health care, which provides a depth of insight that enhances the survey and administrative record.

## 2 Materials and methods

### 2.1 Procedures and participants

As part of a research study funded by the Agency for Healthcare Research and Quality (R01HS028589; Beal, PI), youth-caregiver dyads were enrolled in a 12-month longitudinal study as children (0–17 years old) entered child protective custody and out-of-home care for the first time, with placements in emergency/shelter care, kinship care, licensed foster care, and congregate settings eligible for enrollment. Dyads participating in this study complete surveys every 3 months. For children ages 0–10, caregivers reported children's mental health needs, mental health care access and use, barriers to mental health care, and the impact of mental health on children's daily functioning, using items from the National Survey of Children's Health (7). Caregiver report of children's functioning was collected for all children; self-report data were collected for adolescents 11 and older. When placement changes occurred, youth were retained and new caregivers were enrolled. As children left child protective custody, permanent guardians provided consent to allow the enrolled child to continue participating and also consented to their own participation when appropriate. All study activities, including data extraction and linking described below, were completed with approval from the children's services agency who held custody of children at enrollment and the institutional review board where the research team worked.

At each survey timepoint, youth or caregivers were asked “Did [the child or adolescent] receive all the mental health care that they needed” with response options of “Yes” or “No”. Any adolescent who indicated for themselves or whose caregiver indicated on their behalf that they did not get all needed mental health care on any study survey since enrollment were contacted about participating in a semi structured interview to capture details regarding barriers to and experiences with accessing mental health care. The caregiver with whom the youth was living at the time of the interview was also invited to participate, including biological parents if reunification had occurred before the interview. Caregivers of youth younger than 11 years old participated in the interviews alone. Adolescents aged 11 years old and older were invited to participate in the interview process and could choose to join or not join the caregiver's interview or complete the interview on their own without caregiver participation. Recruitment for interviews continued until thematic saturation was reached. The full qualitative interview guide is provided in Supplementary Table S1. The timeline technique (8) was used to support enhanced recall about mental health care experiences, supported using historical electronic medical record (EMR) and Medicaid data to indicate when mental health care was accessed over the course of enrollment in the study. The recorded interviews were transcribed prior to analysis.

In tandem with study enrollment, EMR and child welfare administrative records where the study occurred were extracted. The participating healthcare system is the only tertiary pediatric healthcare system with emergency and inpatient behavioral health care in the region and houses the foster care clinic where required healthcare for children entering child welfare custody and out-of-home care is provided. EMR were linked with administrative child welfare data to provide information regarding children's services involvement, reasons for entry into out-of-home care, and placement disruptions.

### 2.2 Measures

#### 2.2.1 Sociodemographic and child welfare characteristics

Child age (coded as 0 to 2 years, 2 to 10 years, 10 years and older), gender (male, female, transgender or non-binary), race (White, Black/African American, American Indian or Alaska Native, Asian, Middle Eastern, Native Hawaiian or Pacific Islander, Unknown, Other), ethnicity (Hispanic, Non-Hispanic, Other/Unknown) were extracted from the EMR. Foster care placement details, including number of lifetime placements and placement type (i.e., foster home, kinship, group home, independent living, adoptive placement) at each time point, were extracted from child welfare records. Caregivers self-reported their age, gender (male, female, transgender or non-binary), race (White, Black/African American, American Indian or Alaska Native, Asian, Middle Eastern, Native Hawaiian or Pacific Islander, Other), and ethnicity (Hispanic, Non-Hispanic, Not reported).

#### 2.2.2 Mental health concerns

At each survey timepoint, youth or caregivers were asked, “During the past [12 months (baseline)/ 90 days (follow-ups)], was there any time when [CHILD] needed mental health care or counseling?” with response options of “Yes” or “No”. A response of yes was used to indicate identified mental health needs for analyses. Adolescents and their caregivers each completed PROMIS short forms measuring anxiety and depression (9, 10). HealthMeasures Scoring Service was used to calculate T-scores (mean = 50, SD = 10) for each measure, with higher scores indicating greater symptoms. Caregivers also completed the 20-item Problem Severity subscale from Ohio Scales for Youth, which assesses youth externalizing, internalizing, and conduct problems. Items are rated on a 6-point scale (0 “not at all” to 5 “all the time”) and summed (range 0 to 100’), with higher scores representing more frequent mental and behavioral health concerns (11). If data were missing for caregiver or adolescent report at baseline, we used the earliest reported scores for anxiety symptoms, depression symptoms, and Problem Severity subscales.

### 2.3 Analytic plan

In the full sample of youth and caregivers with survey data, we identified subgroups of youth based on whether the youth or caregiver reported the child 1) needed mental health counseling at any time point and 2) had received all mental health care needed during the first 12 months in care. Descriptive analyses examined proportions of sociodemographic and child welfare characteristics and the mean and variability in mental health concerns across these subgroups.

Among those who completed qualitative interviews, interview transcripts were cleaned and thematically coded by members of the study team with training in thematic content analysis, with double coding of transcripts to establish reliability and consistency. Transcript coding was completed in Dedoose. After transcript coding, themes were reviewed and summarized by four members of the study team. Finally, an analysis of the timelines created during the interviews was conducted to uncover additional patterns in the events described by adolescents and/or caregivers as they sought mental health treatment. Timelines for youth 2–11 years old and timelines for youth aged 11 years old and older were analyzed separately.

## 3 Results

### 3.1 Overview

Results from the descriptive analysis of surveys will be presented first, followed by the results of the timeline analysis that was informed by individual participants’ survey responses and administrative data, and finally the results of the qualitative analysis from the interviews conducted for a subset of participants who completed survey data.

### 3.2 ACUITY survey results

There were *N* = 201 children at enrollment (*n* = 123 under the age of 11, caregiver report data available; *n* = 78 adolescents 11 and older, self-report data available plus *n* = 12 caregivers who agreed to participate in the study with the adolescent). Most (*n* = 196, 97.5%) responded to questions regarding youth mental health needs. There was a 91% retention in survey participation at 12 months.

#### 3.2.1 Mental health need prevalence

Among all youth with caregiver- and/or youth-reported data (*n* = 196), 54% had an identified need for mental health services during the first 12 months in out-of-home care, which was typically first identified upon entry to care (52% of caregivers; 71% of adolescents). Among youth with an identified mental health need (*n* = 106), 89% reported receiving all needed mental health care at least once during the first 12 months in care, though when needs were met varied.

#### 3.2.2 Patterns of reported mental health needs over time

For 32% percent of youth, mental health service needs were consistently met, whereas a persistent need for mental health services was identified across all time points for 13% of youth. The remaining youth had changes in their reported need for mental health services over time: mental health service need changed from unmet to met for 16% of youth, changed from met to unmet for 10% of youth, and varied over time for 25% of youth (Supplementary Figure S1). Changes in reported needs rarely followed a change in placement: 16% of changes in caregiver-reported child mental health service needs followed a placement change, and for adolescent-report it was even lower (10%).

#### 3.2.3 Sociodemographic characteristics across mental health need patterns

Children in the 2–10-year-old age range and those in foster care placements had higher percentage of reported unmet mental health needs compared to adolescents and those in kinship placements. Furthermore, there was a higher percentage of Black caregivers among those who reported mental health concerns for the child and among those who always reported that the child's mental health needs were consistently met. With respect to differences in mental health symptoms, youth whose needs changed to unmet over time had the lowest caregiver reported Problem Severity Subscale scores, whereas youth whose needs changed from unmet to met over time had the highest caregiver reported Problem Severity Subscale. Caregiver reported anxiety and depression symptoms were similar across groups, whereas adolescents whose mental health needs changed from met to unmet had the lowest self-reported anxiety and depression symptoms scores (Tables 1, 2). Other sociodemographic characteristics were comparable across groups.

**Table 1:** Child characteristics by reported mental or behavioral health needs over course of longitudinal survey collection.

Child characteristics	Denied mental or behavioral health needs (*n* = 90)		Endorsed mental or behavioral health needs (*n* = 106)		Endorsed mental or behavioral health needs (*n* = 106)									
					Needs consistently met (*n* = 35)		Needs consistently unmet (*n* = 14)		Needs changed from unmet to met (*n* = 17)		Needs changed from met to unmet (*n* = 11)		Needs met variable over time (*n* = 27)	
	n/m	%/sd	n/m	%/sd	n/m	%/sd	n/m	%/sd	n/m	%/sd	n/m	%/sd	n/m	%/sd
Child age														
<2 years	29	32%	0	0%	0	0%	0	0%	0	0%	0	0%	0	0%
2–10 years	24	27%	61	58%	18	51%	8	57%	8	47%	9	82%	18	67%
10 + years	37	41%	45	42%	17	49%	6	43%	9	53%	2	18%	9	33%
Child gender														
female	45	50%	61	58%	22	63%	11	79%	9	53%	5	45%	13	48%
male	43	48%	44	42%	12	34%	3	21%	8	47%	6	55%	14	52%
nonbinary/transgender	2	2%	1	1%	1	3%	0	0%	0	0%	0	0%	0	0%
Child ethnicity														
Hispanic	8	9%	4	4%	0	0%	4	29%	0	0%	0	0%	0	0%
Non-Hispanic	79	88%	101	95%	35	100%	10	71%	16	95%	11	100%	27	100%
unknown	3	3%	1	1%	0	0%	0	0%	1	6%	0	0%	0	0%
Child race														
American Indian	0	0%	1	1%	0	0%	1	7%	0	0%	0	0%	0	0%
Asian	0	0%	2	2%	0	0%	1	7%	0	0%	0	0%	1	4%
Black	47	52%	52	49%	18	51%	3	21%	9	53%	7	64%	13	48%
Multi-race	12	13%	13	12%	5	14%	2	14%	2	12%	1	9%	3	11%
Other	3	3%	1	1%	0	0%	1	7%	0	0%	0	0%	0	0%
White	28	31%	37	35%	12	34%	6	43%	6	35%	3	27%	10	37%
Placement type														
Foster home	48	53%	58	55%	18	51%	9	64%	6	35%	8	73%	15	56%
Group home	1	1%	4	4%	1	3%	0	0%	2	12%	0	0%	1	4%
Independent living	1	1%	2	2%	0	0%	0	0%	0	0%	2	18%	0	0%
Kinship	37	41%	42	40%	16	46%	5	36%	9	53%	1	9%	11	41%
Adoptive	3	3%	0	0%										
Had a placement change in 12 mos post enrollment	40	44%	51	48%	19	54%	4	29%	7	41%	6	55%	14	52%
Num placement changes	1.23	0.58	1.39	0.63	0.77	0.84	0.29	0.46	0.47	0.62	0.91	1.04	0.78	0.93
Adolescent report														
Anxiety symptoms (T-scores)	48.1	12.4	53.3	12.7	54.6	12.0	54.2	8.9	52.4	15.9	41.4	11.1	54.6	12.7
Depression symptoms (T-scores)	54.1	11.4	57.1	11.3	56.8	12.0	52.3	13.6	59.5	10.6	47.4	17.3	58.8	10.2

Out of those who endorsed mental or behavioral health needs, *N* = 2 were excluded from the analysis of needs met over time given inconsistent reports between adolescent and caregiver.

**Table 2:** Caregiver characteristics by reported youth mental or behavioral health needs over course of longitudinal survey collection.

Caregiver characteristics	Denied mental or behavioral health needs (*N* = 57)		Endorsed mental or behavioral health needs (*N* = 75)		Endorsed mental or behavioral health needs (*N* = 75)									
					Needs consistently met (*N* = 21)		Needs consistently unmet (*N* = 12)		Needs changed from unmet to met (*N* = 9)		Needs changed from met to unmet (*N* = 9)		Needs met variable over time (*N* = 22)	
	n/m	%/sd	n/m	%/sd	n/m	%/sd	n/m	%/sd	n/m	%/sd	n/m	%/sd	n/m	%/sd
Caregiver age	45.2	12	44.7	11.5	46.3	11.7	40.8	15.07	45.2	12.90	52.2	6.96	40.8	9.0
Caregiver type														
Foster parent	39	68%	43	57%	11	52%	6	50%	3	33%	8	89%	13	59%
Kinship	18	32%	32	43%	10	48%	6	50%	6	67%	1	11%	9	41%
Caregiver gender														
female	47	82%	73	97%	21	100%	10	83%	9	100%	9	100%	22	100%
male	8	14%	1	1%	0	0%	1	8%	0	0%	0	0%	0	0%
non-binary/transgender	2	4%	1	1%	0	0%	1	8%	0	0%	0	0%	0	0%
Caregiver ethnicity														
Hispanic	0	0%	1	1%	0	0%	1	8%	0	0%	0	0%	0	0%
Non-Hispanic	52	91%	74	99%	21	100%	11	92%	9	100%	9	100%	22	100%
unknown	5	9%	0	0%										
Caregiver race														
Black	17	30%	35	47%	14	67%	3	25%	4	44%	5	56%	7	32%
More than one race	0	0%	3	4%	0	0%	0	0%	0	0%	2	22%	1	5%
Other	0	0%	1	1%	0	0%	1	8%	0	0%	0	0%	0	0%
White	40	70%	36	48%	7	33%	8	67%	5	56%	2	22%	14	64%
Caregiver report of child mental health														
Problem severity subscale	51.0	13.4	54.3	11.4	14.8	11.29	20.3	14.14	22.7	19.0	6.5	4.9	17.8	11.0
Anxiety symptoms (T-scores)	47.9	10.5	53.5	10.2	51.9	10.6	58.3	6.6	50.1	12.6	52.9	14.7	57.8	10.4
Depression symptoms (T-scores)	9.0	7.8	16.7	13.1	50.9	10.7	59.1	9.6	47.7	9.3	49.5	10.8	57.2	10.0

For youth who had multiple caregivers, these descriptives apply to the first caregiver who responded to surveys. Out of those who endorsed mental or behavioral health needs, *N* = 2 were excluded from the analysis of needs met over time given inconsistent reports between adolescent and caregiver.

### 3.3 Timeline analysis

Forty-three child-caregiver dyads who reported in surveys that they did not receive all the mental health services they needed at any time since enrollment were invited to participate in interviews. Of those asked to participate, 24 did not respond to outreach and 19 completed the interview (*n* = 9 caregivers of children <11 years old, *n* = 10 adolescents 11 years old or older with and without caregivers). See Supplementary Table S2 for participant demographics.

There were different pathways to how youth accessed services over time, similar to what was noted in the survey data. Most of the youth (*n* = 12) did not receive mental health care services prior to their most recent placement, and many (*n* = 16) received mental health services while in out-of-home placement. At the time of the interview, *n* = 11 felt that they were receiving all the mental health services they needed; given that only those who reported at least once that they were not receiving all needed mental health services were invited to participate in the interview, these 11 participants represents a sample whose needs changed from unmet at least once in the past to met. The remaining 8 dyads reported unmet mental health needs at the time of interview, representing a sample of youth with persistent unmet mental health needs or variably met mental health needs over time. Of note, caregivers of youth younger than 11 years old were most likely to report unmet mental health needs at the time of the interview (*n* = 6). Across the 19 interviews, *n* = 8 experienced interruptions in services, indicating variable access to services over time (Supplementary Figure S1). Delays in accessing mental health treatment were very common, occurring for 14 youth.

**Figure 1:**
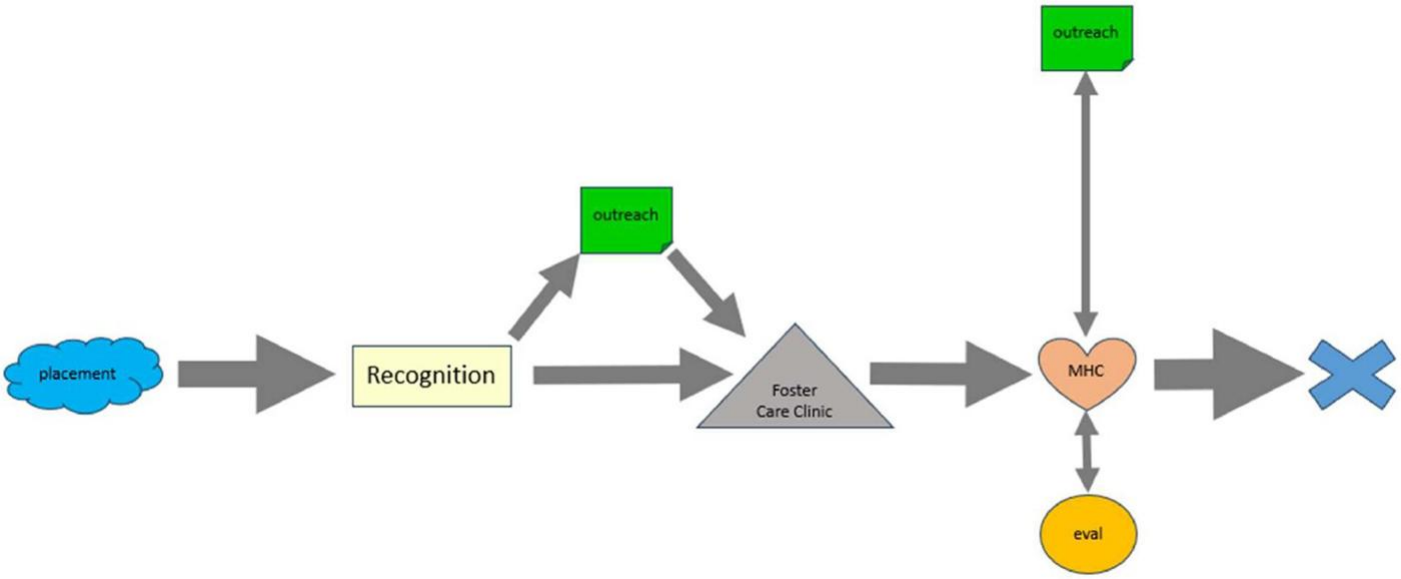
Timeline analysis of interviews with caregivers regarding their experiences seeking mental health care services for the youth in out-of-home placement and younger than 11 years old in their care. Symbols in this figure represent different touchpoints while seeking mental health care. “Placement” refers to when youth were placed in the current home. “Recognition” refers to when the caregiver recognized the child's mental health care need. “Outreach” represents instances where the caregiver reached out to a caseworker for mental health resources. “Foster Care Clinic” represents mandated medical evaluations for children placed in out-of-home care. “Eval” refers to any mental health assessment without treatment, such as psychological testing. “MHC” refers to mental health care service encounters including therapy, day treatment programs, community psychiatric supportive treatment, medication treatment of mental health diagnosis. Speech therapy was also included; however, this only applied to an individual with primary diagnosis of autism. The “X” refers to the time of the interview. The weight of the arrows correlates to how many individuals followed that pathway. Bi-directional arrow indicates that individuals moved back and forth between the two symbols at least once. Only pathways that represent the experience of at least two individuals were included in this figure.

Timeline analysis from interviews with caregivers of children younger than 11 years old revealed that most caregivers (*n* = 8) recognized a need for mental health care services within a month of placement. Of those, caregivers who reported reaching out to their caseworker within a month of recognizing their need accessed mental health resources earlier (Range=2–4 months to starting services, *n* = 3) than those who did not reach out to their caseworkers within a month (Range=5–16 months, *n* = 3). One interviewee reported never accessing services due to not having consent from biological parents. Another interviewee was able to continue previously existing mental health resources without experiencing a gap in care. Only one caregiver reported recognizing mental health care needs more than a month into placement; this child first accessed mental health services 14 months after placement (Figure 1).

**Figure 2:**
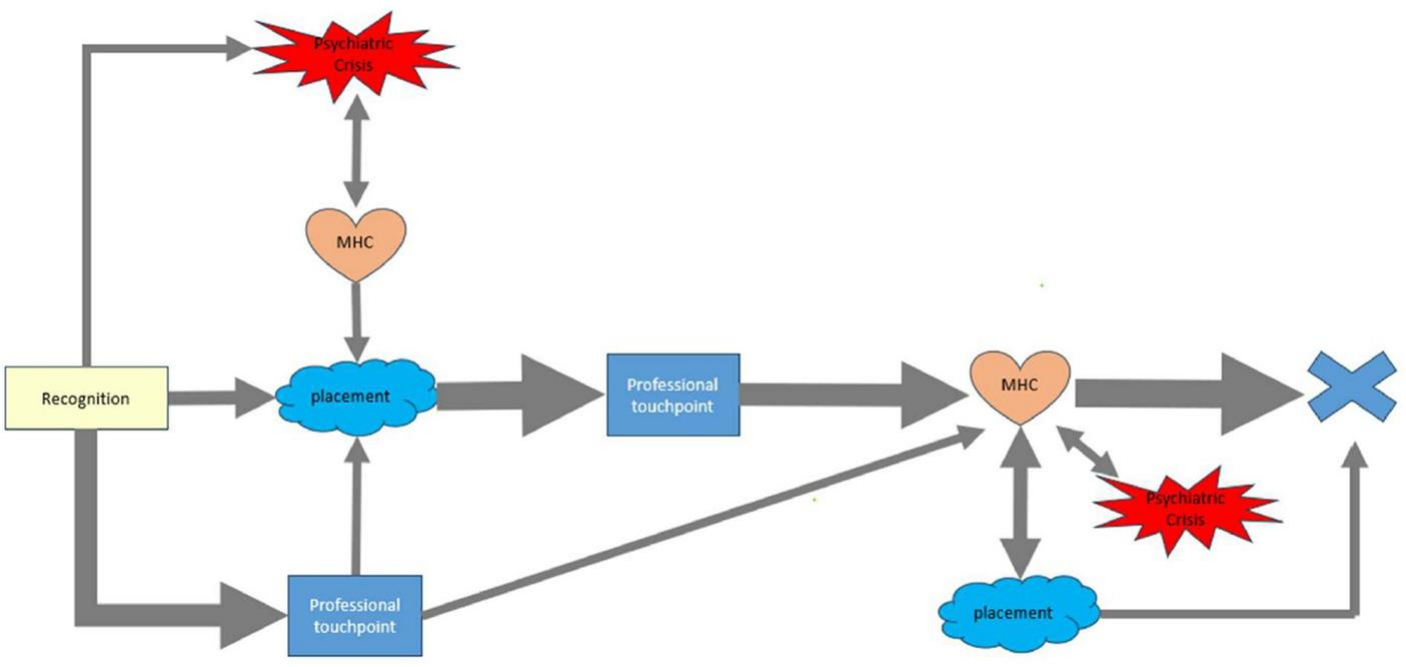
Timeline analysis of interviews with youth and/or their caregivers regarding experiences seeking mental health care services for the youth in out-of-home placement 11 years old and older. Symbols in this image represent different touchpoints while seeking mental health care. “Recognition” refers to when the adolescent's mental health service needs were first identified, either by the caregiver or adolescent. “Placement” refers to when the youth was placed in out-of-home care or experienced a placement change. “Professional touchpoint” includes outreach to caseworker, primary care provider, or mandated medical evaluation for children placed in out-of-home care at the foster care clinic. “MHC” refers to mental health care service encounters including therapy, community psychiatric supportive treatment, and medication treatment of mental health diagnosis. “Psychiatric Crisis” refers to emergency department encounters for mental health or psychiatric inpatient admission. The “X” refers to the time of the interview. The weight of the arrows correlates to how many individuals followed that pathway. Bi-directional arrow indicates that individuals moved back and forth between the two symbols at least once. Only pathways that represent the experience of at least two individuals are included in this figure.

Timeline analysis of adolescent interviews revealed that most adolescents (*n* = 7) reported recognizing mental health care needs before placement into out-of-home care, often years before ever accessing services. Of those, only two adolescents (29%) initiated mental health care services, other than grief counseling, prior to child welfare involvement, and those services were started after emergency department presentations or inpatient admissions for mental health crises. Of the remaining 5 adolescents who recognized need prior to out-of-home care, 4 adolescents initiated services once they entered out-of-home care (range=4–14 months to starting services). Among the 3 adolescents who recognized the need after entry into out-of-home care, 2 received services while in out-of-home care (4 and 5 months to starting services). The third adolescent first recognized a need for mental health services after losing access to services he started as a young child before he could recognize his own mental health needs. He lost these services when he entered out-of-home care and could not reconnect to any services until he was reunified with his parent (Figure 2).

### 3.4 Qualitative interview results

Thematic saturation was reached after 16 transcripts. Participants described both barriers and facilitators to accessing mental health care services. These barriers and facilitators manifested in three primary categories that influenced mental health care service access: Caregiver, youth, and family-of-origin attributes, the mental health system, and other social service systems (e.g., child welfare, Medicaid, school, healthcare) (Tables 3, 4 and Supplementary Table S3).

**Table 3:** Major and minor themes relating to barriers to mental health care services.

Major themes	Minor subthemes	Sample quote
Caregiver, parent, and/or adolescent related barriers (*N* = 16)	• Individual attitudes/perceptions (*N* = 14) • Competing responsibilities/priorities (*N* = 9) • Obtaining biological parent consent (*N* = 5) • Lack of resources (*N* = 2) • Internalized presentation (*N* = 1)	“I was scared that getting mental health services meant that I would be admitted to basically an asylum, because that is what I'm used to hearing whenever mental health is brought up. So, starting out mental health services, I was scared for sure.”
Difficulty communicating information and coordinating services (*N* = 14)	• Communication with caseworkers (*N* = 7) • Information lapses for caregiver (*N* = 6) • Feeling that concerns were not being taken seriously (*N* = 6) • Lack of guidance (*N* = 5) • Communication between systems (*N* = 3)	“So, our case manager, I was telling her that this kid desperately needed therapy and I was asking her for help to get her started in that therapy. Just if she could find somewhere that did not have a bad waitlist and get the people connected to do the intake and stuff like that. And she was not doing it.”
Barriers within mental health services (*N* = 12)	• Service delays (*N* = 8) • Limited options (*N* = 3) • Qualifications (*N* = 2) • Staff turnover (*N* = 2) • Insurance related (*N* = 3)	“[The mental health agency] had called and said that I was still on the waiting list, so we haven't [started mental health services]. I'm still on the waiting list.”
Disruptions related to change of custody or placement (*N* = 8)	• Loss of service with change in location (*N* = 5) • Change to in-home services (*N* = 2) • Changes in insurance (*N* = 2)	“I live in the state of Kentucky, and [the child's old therapist] is in the state of Ohio. It is about a, probably, three-hour drive.”

The number listed next to major and minor themes represents the number of interviews where the theme was present.

**Table 4:** Major and minor themes relating to facilitators to mental health care services.

Major themes	Minor subthemes	Sample quote
Support from non-mental health services (*N* = 18)	• Direction or referrals to services (*N* = 14) • School based mental health resources (*N* = 9) • Support from children's services (*N* = 7) • Integrated care at primary care office (*N* = 4) • Embedded therapy through agency or group home (*N* = 3)	“The only thing that was really helpful, the lady did send me a sheet of a couple.. Before she hung up, she was like, “Hey, I can send you over a list of some places.” That was helpful. The places didn't really work out, but it was helpful in a sense of trying to offer a resource and things like that.”
Characteristics of caregivers and adolescents (*N* = 15)	• Self-advocacy (*N* = 10) • Prior experience (*N* = 7) • Taking initiative (*N* = 7) • Favorable perception of MHC services (*N* = 6)	“I just started calling around and pushing and advocating for all five. There's five of them. So, talking to the schools, advocating for resources.”
Support from mental health care services (*N* = 5)	• MHC provider makes service accessible (*N* = 3) • MHC provider helps to get appropriate consents (*N* = 1) • Insurance coverage (*N* = 1)	“It was the same experience for her where she had to change schools, but the therapist from that old school has continued to see her even though she's not going to that school.”
Continuation of services (*N* = 3)	• Medication (*N* = 2) • Counseling or therapy (*N* = 1) • Information from prior caregiver (*N* = 1)	“I was still enrolled at my current school… so it wasn't hard for me to continue going there [for mental health services].”

The number listed next to major and minor themes represents the number of interviews where the theme was present.

#### 3.4.1 Caregiver, youth, and family-of-origin attributes

Most of the interviews indicated that actions or characteristics of the caregiver or adolescent were helpful in ultimately getting connected to mental health care services. This included advocating for services and taking the initiative to look for and enroll in services.

*“I had to confront [the school-based therapists]. Have you been meeting with her at all? Have you been talking to her? Because I actually teach at the same school, so I was a little bit more on top of it because I could see what was going on. So I questioned them and then they definitely said, ‘Oh yeah, we finally talked to the caseworker, so we're going to start seeing her.’ And then they did an intake.” - Kinship caregiver*

Some caregivers sought mental health services early in placement due to their general perception that having mental health care services was important regardless of the child demonstrating a specific need for those services.

*“Well, he lost his dad so I wanted to make sure that they were able to process that and talk out their feelings. Not only did he lose his dad, but he's living here away from his friends, all that stuff. So I mean, it just made sense to me to get them into somewhere to where they would feel safe and be able to talk about things that maybe they couldn't talk to me about.”- Kinship caregiver*

Other caregivers and youth described delaying or not pursuing recommended mental health care due either to concern about stigma or being unsure regarding the appropriateness of services.

*“I was scared that getting mental health services meant that I would be admitted to basically an asylum, because that's what I'm used to hearing whenever mental health is brought up.” - Adolescent*

Some children benefited from being placed with experienced caregivers who were familiar with navigating the system. Caregivers reported that prior experience caring for children with mental health needs made connecting to services easier.

*“I have a daughter who I also have in therapy, and she's been going since ‘22, so I already had a mental health counselor, who my daughter was seeing, so it was fairly easy.”- Kinship caregiver*

For other children, under-resourced caregivers made access to mental health care challenging. Caregivers reported that transportation and other competing responsibilities, such as work or caring for other children, contributed to delays in care.

*“Again, I work full time. I work remotely one day a week, but it was just narrowing down a time that I could actually get her to therapy.”- Kinship caregiver*

Finally, connections to the family of origin served as both a barrier and a facilitator to mental health care access. In several interviews, not having consent from the biological parent prevented or delayed mental health services. For instance, in the state where this study took place, family of origin consent is required for psychiatric medications when a child is in temporary custody; therefore, psychotropic treatment may be delayed while awaiting consent. Others reported mental health service disruptions between changes of custody or placement, in some instances due to insurance changes.

*“I was battling with insurance, with family services and so on. I was battling with my work. Because after I received custody, they cut the kids off of their insurance, they cut them off of everything, so I had to battle and go start all over with everything for insurance.”- Kinship caregiver*

Biological parents can also recognize shifts in their children's mental health symptoms, which can support better access to care. The biological parent of one adolescent shared that the child's mental health need was overlooked due to his internalized presentation while he was in out-of-home care, which delayed services while the child was in care.

*“I mean, like I said, some cases get in faster. I don't feel like [the adolescent] was really showing what they would want to see with somebody that had a mental health case. You know what I mean? He was still maintaining good grades. He still seemed like a happy kid, but I guess the whole picture wasn't being looked at. I feel like that was a barrier, because although he seemed happy and he seemed like he was normal, I would say on the inside with a situation like that would be traumatizing.”- Biological parent*

#### 3.4.2 The mental health service system

Access to services is a pervasive challenge for children in out-of-home care. Long waitlists, limited availability in specific geographic regions, age-appropriate service options, and staff turnover all contributed to youth not receiving care or experiencing delays in care.

*“I think [the challenge] was mostly just trying to find a good place that didn't have a crazy long waitlist.”- Foster caregiver*

Medicaid insurance barriers also delayed access to mental health services.

*“I had to wait until I got his medical card, and I don't recall how long that took to get the medical card.”- Kinship caregiver*

When mental health service systems and providers are designed to address these barriers, it enhances service delivery and accelerates access. Some interviewees shared that mental health care providers made services accessible through flexible scheduling or help obtaining the necessary consents.

*“[The therapist] connects with me. She couldn't meet [the child] one day at school, so she said she'll see her and she saw her this week. She let me know she saw her. So I mean, it's great. She just went to school because we couldn't get into the office.” - Foster caregiver*

Interviewees also mentioned being able to continue pre-existing mental health services as a strength, even when children changed school districts or moved to other foster care agencies.

*“I was still enrolled at my current school..So it wasn't hard for me to continue going there [for mental health services].” - Adolescent*

#### 3.4.3 Other child serving systems

Relationships between caregivers, youth, and caseworkers were a primary factor discussed in both enhancing access to services and a barrier to care. Receiving clear guidance or referrals to services from a caseworker helped to initiate services, as did advocacy from children's services.

*“School systems and things like that as far as mental health goes - they’re much more quick to help you when you have a legal team advocating for you than they are when you're trying to stand up for yourself and your kid.” - Kinship caregiver*

On the other hand, changes in caseworkers, waiting for referrals or consents from caseworkers, and inadequate guidance when pursuing mental health care resulted in delayed access to care.

*“And [the caseworker] said he would put us in touch with some [mental health resources], but it never really happened. And so I kind of had to be the one that continued to push it, even for the school-based services. I was the one that took care of filling out all the paperwork. And then they had sent over [forms to get] consent from him a few times before he finally responded.” - Kinship caregiver*

Some families were not given important information that would have helped with accessing services, including information needed to continue pre-existing services.

*“I had asked the caseworker a couple of different times [about how to follow up with pre-existing services]. And he was like, “Well, you can just call and figure it out.” Well, he never gave me information on how to contact the people.” - Kinship caregiver*

Mental health service systems are often interconnected with other social service systems, including school, healthcare, and insurance providers. Caregivers recognized that these other systems can help to ensure children get timely access to mental and behavioral health care.

*“The school put her on kind of an expedited list … So they were able to get her into the junior school therapy once she finished her elementary school year.” - Kinship caregiver*

Most commonly, receiving directions or referrals to services from school or healthcare professionals accelerated connections to services. Integrated services in school, primary care, foster care clinics, foster care agencies, and group homes also helped with getting connected to care.

*“I know coming to the [foster care clinic] .. it was nice that you guys were able just to set up appointments for us to do the evaluations,* vs. *me trying to struggle and find a way to get that information.” - Kinship caregiver*

That interdependence of mental health and other service systems can also introduce barriers. In several interviews, caregivers described delays in accessing mental health care due to difficulty sharing information across systems; for example, sharing a Disability Assessment Form (DAF) completed at a mental health agency with the child's caseworker.

*“[The referral agency] said* *..* *since [the child] already had a DAF done they couldn't help us at all. So our county worker, had to hunt down a copy of the DAF that the clinic had done because we didn't even receive a copy of the DAF. We didn't even know her diagnoses or anything. We just knew one existed” - Foster caregiver*

Similarly, mental health services connected to school systems or specific foster care agencies can lead to challenges with maintaining mental health services with changes in placement.

*“And under their licensing, I couldn't have two therapists at the same time. So I went with just the group home one and well, as I said, they quit after a while.”- Adolescent*

## 4 Discussion

This study explored the experiences of children entering out-of-home placement and their caregivers as they sought mental health services. Using survey data collected from youth and caregiver dyads over the course of a year, subgroups were described based on mental and behavioral health service needs and when those needs were met over time. Furthermore, the timeline and thematic analyses of qualitative data collected from interviews with youth-caregiver dyads provided additional information on how these dyads navigated the process of connecting to care.

Survey data showed that most youth entering out-of-home care have mental health needs, consistent with existing data (1, 2). Mental and behavioral health needs were most frequently identified on baseline surveys, especially among adolescent survey respondents. This is consistent with the timeline analysis finding that most adolescents had identified mental health needs prior to entering out-of-home care, and most caregivers identified mental health needs within the first month of placement.

Most survey respondents reported that youth mental health needs were inconsistently met over time, which is similar to what was noted in the interviews. Many interviewees reported ongoing unmet mental health service needs and described disruptions in mental health services over time. This demonstrates the importance of repeated assessments of service engagement over time, even for those already connected to services. There was a higher percentage of youth in the 2–10 years age range in the “Needs Changed from Met to Unmet” and “Needs Variable Over time” groups. Consistently, in qualitative analysis, most caregivers of youth in this age group reported ongoing mental health care gaps. Prior research has shown that children younger than 12 years old access mental health services at a lower rate than adolescents, raising concerns for systemic barriers that may limit access to mental health care in this population (12). This may also be because of increased mental health symptoms among older adolescents, especially those with child welfare involvement (3). Consistently, our findings indicate that children and pre-adolescents with mental health concerns may be particularly vulnerable to changes in mental health service needs and may require special attention when considering interventions to improve access.

There were also notable descriptive differences in the caregiver characteristics among youth with different patterns of mental and behavioral health needs. There was a higher percentage of youth placed with foster caregivers in the “Needs changed from met to unmet” group. Interestingly, prior research has shown that placement type is a predictor of mental health care utilization, with children in foster care placement more likely to utilize mental health care services than those in kinship placement (13). This discordance in findings could be due to interpretation of the survey item. Whether or not mental health needs were “met” could be a better reflection of perceived service adequacy rather than service utilization. For example, while children in foster care placements are more likely to utilize mental health services, we do not know if these services are perceived as meeting the child's health care needs; it is possible that children in foster care are more likely to have mental and behavioral health needs that are difficult to adequately address with available services. Of note, many of the interview respondents who reported continued unmet mental health service needs were connected to mental health services but did not feel that those services sufficiently addressed their mental health concerns. Caregiver perception of service adequacy appears to be an important factor to mental health service access. The thematic and timeline analyses found that caregivers play a unique role in ultimately connecting a child to mental health services, particularly in how proactively they pursue services and follow up on recommendations. This finding is consistent with the idea that caregivers can act as “gate keepers” to mental health services (14). Caregivers may be more likely than the youth in their care to report concerns about the youth's mental health. Of note, 74% (*n* = 14) of caregiver participants who were caring for an adolescent (*n* = 19) reported mental health concerns for the adolescent in their care, while 51% (*n* = 41) of adolescent participants (*n* = 81) self-reported mental health concerns. If caregivers are more likely to report mental health concerns than youth, this may account for both survey and qualitative data indicating more unmet mental health needs in the 2–10-year age group – as all survey and interview responses for this age group were from caregivers. However, this consideration should be balanced with systemic barriers that may also be contributing to this finding, as described above. Regardless of cause, these findings indicate that gaps in mental health access for children remain (15), which are likely because of pediatric provider shortages that are especially prominent among children experiencing adversity (16).

Furthermore, baseline caregiver reported Problem Severity Subscale scores were lower in the “Needs changed from met to unmet” and the highest average baseline Problem Severity Subscale score was found in the “Needs changed from unmet to met” group. On the other hand, caregiver reported anxiety and depression scales were relatively stable across groups. Given that the Problem Severity Subscale score includes externalizing symptoms, this finding is consistent with previous evidence that externalizing symptoms are more likely to prompt mental health service utilization (17, 18). Externalizing symptoms prompting mental health service use was observed during adolescent timeline and qualitative analysis. Only adolescents with behaviors requiring emergency department level of care connected to mental health care services prior to out-of-home placement. Furthermore, during the interview, one biological parent felt that her child's mental health needs went unmet due to the internalized nature of symptoms.

These findings should be interpreted in the context of well documented structural inequities that exist within the child welfare and mental health systems (19, 20). It is important to consider the ways that structural racism and poverty affect disproportionality in child welfare and disparities in mental health outcomes and access as they likely play a role in patterns observed between groups within our survey responses. For example, research indicates that Black and Hispanic youth are less likely to receive mental health care, even among those who have experienced trauma and chronic stress (21). Low mental health service utilization in this population is likely multifactorial, but medical mistrust, a result of historic and ongoing racism in health systems, may play a role, specifically in how caregivers may report mental health care needs or seek mental health services for the child in their care. However, given the study's design, no specific conclusions can be made regarding the ways equity and structural racism affected outcomes.

Another important finding from this study is the important role supporting systems have in the acquisition of mental health services. School-based mental health services play a particularly important role in mental health care access, with almost half of the interviewees accessing mental health through schools. Given high utilization of school based mental health services, this is an important area for additional investigation and expansion to ensure that high quality, evidence-based services can continue to be provided in in these settings. Furthermore, many respondents shared that simply having a referral to services from a professional was helpful, offering a straightforward intervention that can be implemented in many settings. Relationship building and sharing information between systems would help facilitate these referrals. For instance, integrated mental health services in primary care clinics and child welfare services allow for easy and direct referral processes. Regular communication between systems can also help establish referral pathways that are easier for the child and caregiver to navigate. These findings highlight the importance of continuing to strengthen mental health presence across child serving systems.

Furthermore, while systemic challenges, including limited communication between child welfare and mental health systems, have previously been documented as a barrier to stably accessing mental health care (4–6, 22), this study uniquely highlights the ways caregivers feel unsupported and lost in this process. A family-based mental health navigator is an intervention that shows promise in ameliorating some of these challenges. The navigator model utilizes an in-person navigator and adjunctive digital health technology to improve care coordination and monitoring of mental health service needs for youth (23). Such an intervention would be ideally suited to address the challenges in communicating and coordinating services in mental health and broader social service systems, in addition to the individual-level challenges households face, identified in the qualitative analysis. For instance, many caregivers identified difficulty communicating with the case worker as a barrier to mental health care services; a navigator could facilitate identification and referrals to services in place of the caseworker, thus increasing time for the caseworker to address other aspects of the child's care. A mental health navigator may also bridge gaps for children without a caregiver advocate, a role this study found to be particularly important in the process of accessing services.

### 4.1 Limitations

The survey data presented was descriptive and meant to provide additional context to the qualitative data in this study. Therefore, interpretation of the survey data alone is limited. Generalizability of findings from the qualitative analysis is limited due to several factors including small sample size and underrepresentation of Black youth and youth in foster care family placements. Furthermore, the data from surveys and recruitment for interviews was based on self-report data. Therefore, the findings of this study cannot be applied to children whose mental health needs may be unidentified.

### 4.2 Conclusions and future directions

This study offers unique insight into the barriers and facilitators to mental health care services for child welfare involved youth. Specifically, this study highlights the lived experience of youth and their caregivers as they seek mental health care, which has not been a focus in existing literature. Furthermore, utilization of the timeline technique and timeline analysis, has not previously been done in studies examining this issue. The timeline analysis, in addition to the longitudinal survey data, adds a deeper understanding of the experiences and processes described. These analyses allowed for two important findings: 1) mental health care utilization by youth in foster care is largely driven by their caregivers and 2) many youths in out-of-home placements benefit from integrated mental health resources. These findings illustrate the need for interventions that decrease reliance on caregivers to initiate mental health services; for instance, a family-based mental health navigator can help improve access to services for youth without caregiver advocates. Similarly, integrated mental health creates referral pathways that are easy for youth and caregivers to navigate. Efforts to improve mental health care access should focus on strengthening and expanding integrated services.

Future research should include adolescents and caregivers without self-reported mental health concerns to better understand how mental health needs are identified and, subsequently, what encourages or discourages individuals from seeking care. Furthermore, implementation and evaluation of interventions meant to improve mental health access will be important – for instance implementation of a navigator model for families seeking mental health care (23). Moving forward, partnerships between mental health care systems, child welfare systems, and caregiver-child dyads will be essential to address mental health care access challenges and prevent further mental health escalation and placement instability.

## Data Availability

The raw data supporting the conclusions of this article will be made available by the authors, upon request and without undue reservation.
