# Supplementary figures and images for: Barriers and facilitators to mental health care experienced by youth involved in child welfare and their caregivers
Source: Front Pediatr. 2026 Apr 20;14:1763516. doi: 10.3389/fped.2026.1763516 (PMC13136629; doi:10.3389/fped.2026.1763516)

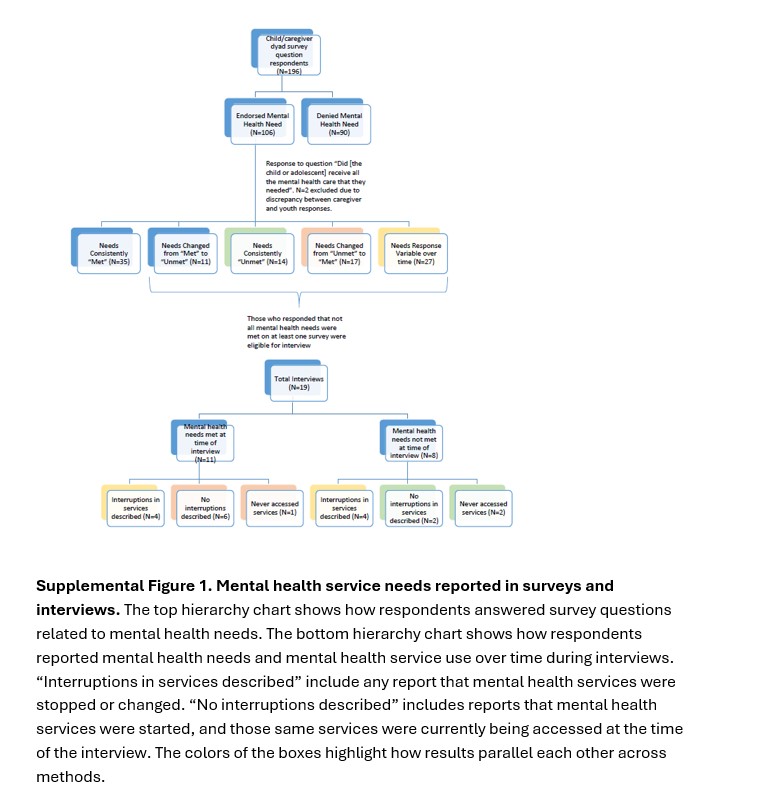

Supplement: Supplementary file 1 [file Image1.jpeg]
