# Supplementary material for: Barriers and facilitators to mental health care experienced by youth involved in child welfare and their caregivers
Source: Front Pediatr. 2026 Apr 20;14:1763516. doi: 10.3389/fped.2026.1763516 (PMC13136629; doi:10.3389/fped.2026.1763516)
Supplement: Supplementary file 2 [file Table1.docx]

| **Supplemental Table 1. Semi-structured interview guide for interviews conducted with caregivers and or youth regarding their experiences accessing mental health services for the youth in out-of-home placement** | | | |
| --- | --- | --- | --- |
| **Question Number** | **Theme** | **Question** | **Follow up Prompts (if needed)** |
| 1 | Initial Placement | When did [INSERT NAME OF CHILD] start living with you? |  |
| 2 | Initial Placement | What, if anything, do you know about the mental health services, like therapy or medication management, [INSERT NAME OF CHILD] was receiving before they came into your home? | What services were they getting? When they came to your home, did those services continue or stop? |
| 3 | Initiating Mental Health Care | When did you notice [INSERT NAME OF CHILD/you] might benefit from mental health care? This can include a professional telling you that [INSERT NAME OF CHILD/you] might benefit from mental health care even if you might not have known or felt like mental health services were needed at that time. [add date to timeline] |  |
| 4 | Initiating Mental Health Care | What happened next? | An evaluation with child protection services, seeing a medical provider, calling mental health services. Continue to probe about the next event until mental health services are obtained. [add dates to timeline] |
| 5 | Initiating Mental Health Care | Tell me about your experience initiating mental health care. | What made it easy? What made it difficult? |
| 6 | Experience with Mental Health Care | What mental health appointments with different mental health professionals should be marked on this timeline? This includes providers that [INSERT NAME OF CHILD/you] might have seen for talk therapy or medication management. |  |
| 7 | Experience with Mental Health Care | [Pointing to changes in providers on timeline] what led to those changes? |  |
| 8 | Experience with Mental Health Care | Tell me a little bit about your experiences with these services. |  |
| 9 | Experience with Mental Health Care | Do you think [INSERT NAME OF CHILD/you] received mental health services too soon, right when they needed them, or too late? Why? | Which mental health services, if not all, does your response apply to specifically? |
| 10 | Experience with Mental Health Care | Do you feel like [INSERT NAME OF CHILD/you] is/are getting all the mental health services they/you need now in your home? | If not, why? |
