# Supplementary material for: Barriers and facilitators to mental health care experienced by youth involved in child welfare and their caregivers
Source: Front Pediatr. 2026 Apr 20;14:1763516. doi: 10.3389/fped.2026.1763516 (PMC13136629; doi:10.3389/fped.2026.1763516)
Supplement: Supplementary file 3 [file Table2.docx]

| **Supplemental Table 2. Demographics of youth and caregivers recruited and interviewed for qualitative analysis** | | | | | | |
| --- | --- | --- | --- | --- | --- | --- |
| **Child Demographics** | | | | | | |
|  | | | | **Total Sample**  **N = 201** | **Recruited to Interview**  **N = 43** | **Interviewed**  **N = 19** |
| Age at enrollment, years – M (SD) | | | | 8.57 (5.45) | 10.69 (4.06) | 9.63 (4.05) |
| **Gender N (%)** | | | | | | |
|  | Female | | | 110 (55%) | 28 (65%) | 12 (63%) |
|  | Male | | | 88 (44%) | 15 (35%) | 7 (37%) |
|  | Transgender or Non-Binary | | | 3 (1%) | 0 (0%) | 0 (0%) |
| **Race/Ethnicity N (%)** | | | |  |  |  |
|  | Black or African American | | | 102 (51%) | 19 (44%) | 7 (37%) |
|  | White | | | 64 (32%) | 15 (35%) | 10 (53%) |
|  | Other | | | 6 (3%) | 2 (5%) | 0 (0%) |
|  | More than one race | | | 29 (14%) | 7 (16%) | 2 (11%) |
| **Ethnicity N (%)** | | | | | | |
|  | Non-Hispanic | | | 186 (93%) | 40 (93%) | 19 (100%) |
|  | Hispanic | | | 12 (6%) | 3 (7%) | 0 (0%) |
|  | Not reported | | | 3 (1%) | 0 (0%) | 0 (0%) |
| **Placement at baseline N(%)** | | | |  |  |  |
|  | | Foster Home | | 103 (51%) | 20 (47%) | 5 (26%) |
|  | | Kinship Placement | | 90 (45%) | 19 (44%) | 11 (58%) |
|  | | Group Home | | 5 (2%) | 3 (7%) | 3 (16%) |
|  | | Independent Living | | 3 (1%) | 1 (2%) | 0 (0%) |
| **Number of placement changes N (%)** | | | |  |  |  |
|  | | No placement change | | 171 (85%) | 34 (79%) | 14 (74%) |
|  | | One placement change | | 24 (12%) | 6 (14%) | 3 (16%) |
|  | | Two or more placement changes | | 6 (3%) | 3 (7%) | 2 (11%) |
| **Caregiver Demographics** | | | | | | |
|  | | | | **Total Sample**  **N = 135** | **Recruited to Interview**  **N = 22** | **Interviewed**  **N = 16** |
| Age at enrollment, years – M (SD) | | | | 44.80 (11.73) | 40.81 (12.82) | 46.09 (14.31) |
| **Gender N(%)** | | | |  |  |  |
|  | | | Female | 121 (90%) | 21 (95%) | 14 (88%) |
|  | | | Male | 9 (7%) | 0 (0%) | 0 (0%) |
|  | | | Transgender or Non-Binary | 3 (2%) | 1 (5%) | 1 (6%) |
|  | | | Not reported | 2 (1%) | 0 (0%) | 1 (6%) |
| **Race N(%)** | | | |  |  |  |
|  | | | Black or African American | 53 (39%) | 5 (23%) | 4 (25%) |
|  | | | White | 76 (56%) | 15 (68%) | 10 (62%) |
|  | | | Other race | 1 (1%) | 1 (5%) | 0 (0%) |
|  | | | More than one race | 3 (2%) | 1 (5%) | 0 (0%) |
|  | | | Not reported | 2 (1%) | 0 (0%) | 2 (12%) |
| **Ethnicity N(%)** | | | |  |  |  |
|  | | | Non-Hispanic | 127 (94%) | 21 (95%) | 14 (88%) |
|  | | | Hispanic | 1 (1%) | 1 (5%) | 0 (0%) |
|  | | | Not reported | 7 (5%) | 0 (0%) | 2 (12%) |
| **Caregiver type N(%)** | | | |  |  |  |
|  | | | Foster caregiver | 83 (61%) | 12 (55%) | 3 (19%) |
|  | | | Kinship caregiver | 52 (39%) | 10 (45%) | 11 (69%) |
|  | | | Biological parent | 0 (0%) | 0 (0%) | 2 (12%) |
| Interview participants were recruited based on survey responses in longitudinal survey collection indicating that the child was not getting all needed mental health care. “Total Sample” represents the total number of enrolled longitudinal survey participants. “Recruited to Interview” represents those that received any outreach from clinical research coordinators to participate in interview based on their survey response that they were not getting all needed mental health services. “Interviewed” includes those that consented to and participated in the interview. | | | | | | |
