# Supplementary material for: Barriers and facilitators to mental health care experienced by youth involved in child welfare and their caregivers
Source: Front Pediatr. 2026 Apr 20;14:1763516. doi: 10.3389/fped.2026.1763516 (PMC13136629; doi:10.3389/fped.2026.1763516)
Supplement: Supplementary file 4 [file Table3.docx]

| **Supplemental Table 3. Number of interview participants in each endorsed mental or behavioral health needs group from survey responses with sample quotes describing overall experience accessing mental health services.** | |
| --- | --- |
| Needs consistently unmet (N=4) | |
|  | *“Just downright awful, but I think that it's a mixture of our location. We live out in [a rural area] and there's one small ER. There's nothing really out here. There aren't any mental health professionals other than, like I said, in the school, they have [school based therapy]. That's it. If you want to go to therapy, I don't even know how far out it is. And then having to work full time and then having to take off work because limited schedules, limited therapists, things like that, that kind of just add to the mixture. And then when you add that you have to wait on other people to consent and you're waiting on someone to fill out a 20-page consent form, they take their time with it. So it's just a mixture of a lot of different things that kind of go into the pot and then it's like nobody turned the stove on. It's just sitting there.”- Kinship caregiver* |
| Needs changed from unmet to met (N=3) | |
|  | *“So let me keep it real with you. I came out of [juvenile detention] when I came here, so I went from receiving zero mental health to at least some to keep me stable. So it felt like a big jump, even though it might've not been that much. But yeah, that's really it. I went from basically no mental health services to that, so it was a nice transition at least.” – Adolescent* |
| Needs changed from met to unmet (N=3) | |
|  | *“…honestly, because he's only in the temporary custody of the county, mom still has that say so, so he still hasn't been set up with any services sadly, because mom won't agree to them.” – Foster parent* |
| Needs variable over time (N=9) | |
|  | *“Red tape and staffing things [make getting services difficult]. Just staff turnover, staff changes, and everyone having different opinions on what will help the kid and what won't, instead just from the start being like, ‘Hey, this kid has trauma. Let's get this kid therapy’. Or people being out for surgeries. Or just between all the stupid red tape and paperwork that some of it's like, ‘Why do you actually need her Social Security?’. Silly little stuff like that. Getting consent spelled out, things like that. And then from that, to just the waiting on all the steps to happen. Even waiting to hear that [mental health agency] doesn't have a therapist. That's two weeks that we waited there when we weren't looking into something else. Just all the waiting and the silly red tape that most of it doesn't even seem that necessary and the staffing stuff [made getting services difficult].” – Foster parent* |
| During the semi-structured interview, participants were asked to “Tell me about your experience initiating mental health care” with optional follow prompts of “What made it easy? What made it difficult”. Sample responses to this question are shown in this table. These sample responses are matched to the participant’s corresponding mental and behavioral health needs group based on their longitudinal survey responses over time to the question “Did [the child or adolescent] receive all the mental health care that they needed?”. | |
